# A Case Report of Congenital Afibrinogenemia and Literature Review of Management of Post-circumcision Bleeding

**DOI:** 10.7759/cureus.36459

**Published:** 2023-03-21

**Authors:** Imteyaz Khan, Matthew Chow, Shakuntala Chandra, Mark Hiatt

**Affiliations:** 1 Neonatology, Saint Peter’s University Hospital, New Brunswick, USA; 2 Pediatrics, Saint Peter’s University Hospital, New Brunswick, USA

**Keywords:** fibrin glue, post circumcision bleeding, coagulopathy, afibrinogenemia, male circumcision

## Abstract

We present a case of bleeding from circumcision in a full-term newborn male resulting from a rare coagulopathy, congenital afibrinogenemia, and a review of the literature regarding the management of bleeding after circumcision. Bleeding was managed with silver nitrate, suturing, thrombin powder, Arista^TM^ AH (absorbable hemostatic particles; Becton, Dickinson and Company, Franklin Lakes, USA), FFP (fresh frozen plasma), and cryoprecipitate. The Fibrinogen level was less than 30 mg/dl (ref 150-430 mg/dl). The diagnosis of congenital afibrinogenemia was confirmed by a gene test. The baby was found to have a heterozygous pathogenic variant (c.510+1G>T) and a heterozygous likely pathogenic variant (c.1037del) in the *FGA* gene.

## Introduction

Circumcision of a male newborn is one of the oldest surgical procedures known to humanity [1]. It is still performed almost the same way as it was thousands of years ago in some communities [2]. Improvements in medical knowledge and sterile techniques have dramatically reduced complications after circumcision. Severe bleeding after circumcision is rare and usually stops when digital pressure is applied. However, in a few cases, coagulopathy can lead to catastrophic bleeding. Circumcision is typically performed in the United States within 2 to 3 days of birth in the birthing hospital [3]. Most of the time, rare congenital coagulopathies are not detected until this time [4]. Newborns with rare coagulopathies, such as congenital afibrinogenemia, usually present with post-circumcision bleeding [5]. Literature regarding the management of post-circumcision bleeding in newborns with coagulopathy is scant, and more research is needed to develop guidelines for managing post-circumcision bleeding in newborns.

## Case presentation

A bi-racial, three-day-old male newborn, born at 39 weeks in a community hospital, was transferred to our tertiary care neonatal intensive care unit after he suffered uncontrollable bleeding following circumcision. The mother is a 32-year-old G3 P3 T2 Pr0 Ab1 LC2, blood type A positive, syphilis screen non-reactive, HIV negative, Hepatitis B negative, Rubella immune, and GBS (Group B Streptococcus) negative. Although she tested positive for SARS-CoV-2, she was not symptomatic. The infant tested negative for COVID-19. At birth, he was appropriate for gestational age based on a birth weight of 3.4 kilograms with an APGAR score of 8 at one minute and 9 at five minutes. The mother and father do not have a family history of bleeding disorders. The family was not consanguineous. The mother is Caucasian and the father is Hispanic.

The infant was circumcised on day 3 at the request of his mother. After circumcision, the newborn experienced extensive bleeding, which necessitated suturing by a urologist. The urologist also applied a silver nitrate solution to control bleeding. Upon workup, the infant showed elevated PT (prothrombin time), PTT (partial thromboplastin time), and low fibrinogen levels. The infant was transferred to our tertiary care neonatal intensive care unit for pediatric hematology consultation and further treatment. After arriving at our hospital, a physical examination revealed that he had capillary refills of three seconds, pallor, oozing blood from a previous arterial puncture on the left wrist, a hard indurated muscle hematoma on the left thigh caused by a previous intramuscular vitamin K injection, circumcised penis with ventral separation, five-zero chromic sutures, swelling of the glans with silver nitrate wound of the glans, blood oozing through the Surgicel wrapping, and tenderness to palpation. The remainder of the physical examination was unremarkable.

Initial lab results were WBC: 9.2000 per microliter of blood, hemoglobin 10.7 gram per deciliter, and platelets: 278,000 per microliter of blood. PTT: >200.0, PT: >180.0, international normalized ratio (INR): >10.00, fibrinogen level <35, Factor II levels at 1 unit/dl. A pediatric hematologist, a pediatric urologist, a pediatric surgeon, and a pediatric geneticist were consulted. The bleeding circumcision was treated with 5000 units of thrombin powder and twice with Surgicel, but the bleeding continued. Despite the application of Arista^TM^ AH (absorbable hemostatic particles; Becton, Dickinson and Company, Franklin Lakes, USA) to the bleeding circumcision, bleeding continued. Following the administration of fresh frozen plasma (FFP) followed by cryoprecipitate and 20 cc/kg of packed red blood cells, the bleeding stopped. The patient was given three mL/kg FFP every 12 hours and Amicar (aminocaproic acid) 100 mg/kg every six hours was used to stabilize the clots and keep clotting factors at hemostatic levels.

After the blood transfusion, hemoglobin increased to 12.4 mg/dl, which did not decrease further during the remainder of the hospital stay. There was a small wound on the penis with a yellow discharge, likely due to previous silver nitrate application. Following two weeks of applying bacitracin and zinc, every time a diaper was changed, the wound healed. There was no intracranial bleeding. An echocardiogram showed no abnormalities. After day 4, there was no further bleeding. The infant remained hemodynamically stable. PT/INR, PTT, and fibrinogen results were satisfactory, and Factor II activity was 71%. The FFP infusion was discontinued. The results of a repeat factor assay 72 hours after discontinuing FFP are shown in Table 1. A blank space indicates that the factor level was not performed on that day in order to minimize the loss of blood during phlebotomy.

**Table 1:** Blood coagulation factor level with reference range.

	Normal Range	Level on day 3 of life	Level on day 8 of life	Level on day 14 of life	Level on day 16 of life
Prothrombin Time (PT)	9.4-12 seconds	>180	11.6	>180	
Partial Thromboplastin Time (PTT)	22.5-30.1 seconds	>200	27.6	>200	
Internation Normalized Ratio (INR)	0.9-1.1	>10	1.01	>10	
Fibrinogen	150-430 mg/dl	<35	112	41	<30
Factor II	75-127 unit/dl	1	71	65	
Factor VII	66-173 unit/dl		98	104	
Factor VIII	53-131 unit/dl		140	159	
Factor IX	63-112 unit/dl		39	33	
Factor X	72-145 unit/dl		104	100	

Since fibrinogen was low and decreasing, a concern about congenital fibrinogen deficiency was raised suggesting afibrinogenemia or hypofibrinogenemia. Fibrinogen panel and genetic study were sent and were significant for fibrinogen level <30. The genetic studies were sent two weeks after the blood transfusion. A gene test confirmed the diagnosis of congenital afibrinogenemia. The baby was found to have a heterozygous pathogenic variant (c.510+1G>T) and a heterozygous likely pathogenic variant (c.1037del) in the *FGA *gene; the phase of these variants is unknown (cis or trans). The mother was informed that biallelic pathogenic variants in the *FGA *gene are associated with autosomal recessive afibrinogenemia and autosomal dominant/autosomal recessive congenital dysfibrinogenemia. Both variants identified in the baby have previously been reported in individuals with autosomal recessive afibrinogenemia. On day 20, the umbilical cord fell off. There was no bleeding. On day 22, the infant was discharged home.

## Discussion

Approximately one to two out of every one million newborns are affected by congenital afibrinogenemia [6,7]. It is inherited in an autosomal recessive manner. The first description of congenital absence of fibrinogen (afibrinogenemia) was published in 1920 [5]. The cause of congenital afibrinogenemia is a mutation in one of three genes, *FGA*, *FGB*, or *FGG*, located on chromosome 4 (q26-q28). These three genes encode a hexametric glycoprotein called fibrinogen, which contains one part (subunit) for each gene. The fibrinogen protein, which measures about 340 kDa and is synthesized in the liver, serves a variety of functions, including the formation of fibrin clots, non-substrate thrombin binding, platelet aggregation, and fibrinolysis [8]. To prevent excessive bleeding after injury, this protein is critical for the formation of blood clots (coagulation). As a result of an injury, fibrinogen converts into fibrin, the principal protein in blood clots. Blood clots are formed by fibrin proteins attaching to each other and forming a stable network.

The absence of fibrinogen protein causes congenital afibrinogenemia. A premature stop signal is produced when the instructions for making each of the *FGA*, *FGB*, and *FGG *proteins are altered due to mutations in these genes. No protein is functional if it is produced. A missing subunit will result in the fibrinogen protein not being able to assemble, and thus fibrin will not be formed. Therefore, people with congenital afibrinogenemia do not form blood clots in response to injury, resulting in excessive bleeding [6]. Afibrinogenemic patients have undetectable or nearly undetectable levels of fibrinogen (<10 mg/dL; normal 150-430 mg/dL) by activity-based clotting assays and by measurement of immunoreactive fibrinogen. In the absence of consumptive coagulopathy, an unmeasurable fibrinogen level is diagnostic of the condition [9]. Afibrinogenemia is often diagnosed in the neonatal period with 85% presenting with umbilical cord bleeding [5]. Additionally, central nervous system (CNS) bleeding (5%) and bleeding into the skin, gastrointestinal tract, and genitourinary tract are also frequent presentations. Hemarthrosis is the most common form of musculoskeletal bleeding, occurring in 54% of patients. Mucocutaneous, soft-tissue, joint, and genito-urinary spontaneous bleeding, and traumatic or surgical bleeding are the most common clinical symptoms. There is also a report of excessive menstrual bleeding in women. Rarely, thrombosis, poor wound healing, and splenic rupture occur [10,11,12,13].

According to one study, seven (54%) out of 13 pregnancies in six women who had afibrinogenemia ended in spontaneous abortions between six and seven weeks of gestation, thus supporting the requirement of fibrinogen for the implantation of an embryo [14]. Bleeding can be managed with purified plasma-derived concentrates (RiaSTAP® Fibrinogen Concentrate (Human-derived), CSL Behring GmbH, King of Prussia, USA), cryoprecipitates, or fresh frozen plasma. There is a risk of viral transmission, antibody development, and thromboembolic events associated with the use of some of these products. The establishment of registries in Iran, Italy, and North America has led to a better understanding of these disorders. In addition, it has led to an attempt to explore the molecular defects associated with them. As a result of initiatives developed by the United States and other countries, there is hope that safer, more effective products will be developed and licensed [15].

Management of bleeding from circumcision

Male circumcision is one of the oldest operations traced back as early as the stone age, 15,000 BC [1]. In the world today, it is the most common procedure. It is performed for a variety of reasons, including religious, cultural, and social reasons, including medical [16]. Medical benefits are insufficient to justify routine neonatal circumcision for all male newborns, according to the Canadian Paediatric Society and the American Academy of Pediatrics [17,18]. It is estimated that 30 percent of males worldwide are circumcised. A total of nearly 1.3 million newborn males are circumcised during birth hospitalization each year in the United States, which represents 55.8% of all births of males [19]. According to estimates, there are 84.5 million males aged 15 years and older in the United States who have undergone circumcision for non-religious reasons. The United States is the only country where the vast majority of infants at birth are circumcised for non-religious reasons [16]. 

In neonatal and infant circumcisions, the median frequency of any postoperative complications is 1.5%. The most common complications are bleeding, pain, and infection [20]. Most newborns do not experience excessive bleeding after circumcision, but some newborns with previously unknown coagulopathy may experience severe bleeding after circumcision as in our case, where there was no history of bleeding disorders in the family and no consanguinity. Clinical professionals who perform circumcision must be aware of their options to manage bleeding and have protocols and resources in place to prevent life-threatening bleeding, including timely transfer to a tertiary care center. When circumcision is performed at an older age, the risk of severe bleeding increases. A US study found no complications among 98 circumcised boys in the first month of life. However, 30% of boys aged 3-8.5 months had significant postoperative bleeding [21]. 

The severity of post-circumcision bleeding can be divided into three categories in diagnosed hemophilia patients. The same can be applied to newborns who present with post-circumcision bleeding but have not yet been diagnosed with coagulopathy before circumcision. Mild bleeding is defined as oozing that stops spontaneously or with simple management, like digital pressure, and is only treated with factor infusion. Profuse bleeding, penile hematoma, resuturing, and factor replacement over one day are considered moderate bleeding. The definition of a severe bleeding event is one that requires blood transfusions and factor replacements and is considered a life-threatening event [22]. Often, bleeding can be managed conservatively with pressure, silver nitrate, and fibrin glue before undergoing surgery [23]. Coagulopathy, however, can cause severe postoperative bleeding.

**Figure 1:**
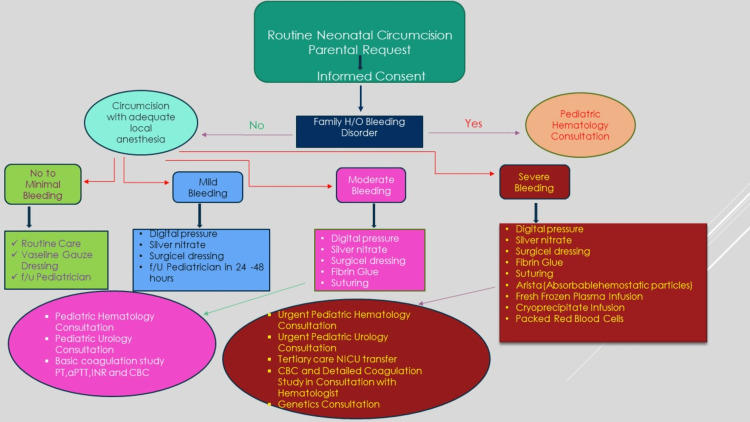
Flowchart for management of post-circumcision bleeding in newborns. H/O: history of; f/u: follow up; CBC: complete blood count; PT: prothrombin time; aPTT: activated partial thromboplastin time; INR: international normalized ratio

A limited number of studies have been conducted on circumcision in patients with coagulopathies. In addition to suturing, there are alternatives such as cyanoacrylate glue and disposable clamps [24]. For patients with coagulopathy diagnosed prior to surgery, several studies have highlighted the importance of preoperative factor replacement. It should be noted that due to the possibility of inhibitor development, some argue against circumcision or recommend bundling the procedure with another procedure. Based on the 2009 European Hemophilia Therapy Standardization Board survey, it was recommended to replace factor 80% to 100% preoperatively and to continue replacement therapy for three to four days after surgery [25]. Those patients with hemophilia who must undergo circumcision due to religious reasons must undergo factor replacement prior to and after the procedure. There has also been evidence that fibrin glue can reduce the amount of recombinant factor replacement that is required (as well as the cost of the treatment) without causing significant changes in bleeding complications [26]. The use of fibrin glue (also known as fibrin sealant) is recommended by some centers for all circumcisions that are performed on known hemophilia patients. Circumcision is followed by bolus injections of factor concentrate every 12 hours. Plasma factor levels are maintained between 50% and 60% for just three days [22]. As a hemostatic agent, fibrin sealant creates a fibrin clot. Human fibrinogen and human thrombin are separately packaged in this product [27]. The mixture transforms fibrinogen into fibrin monomers. Thrombin converts factor XIII to factor XIIIa. XIIIa crosslinks fibrin monomers to a polymer, increasing its resistance to fibrinolytic degradation. As wound healing proceeds, plasminogen activators present in the tissue activate the plasminogen present in plasmin. Soluble fibrin products are produced by fibrinolytic activity. Aprotinin (protease inhibitor) in concentrate retards this process. Also, tranexamic acid (not included in the glue) inhibits fibrinolytic activity and helps to prevent fibrinolysis. In this way, wounds heal without bleeding [26]. We recommend that fibrin glue be easily available in centers that perform a large number of circumcisions and propose the following flow chart for a quick consultation (Figure 1).

## Conclusions

This case demonstrates the risk of catastrophic bleeding post-circumcision in a healthy-term infant with congenital afibrinogenemia. Early use of fibrin glue may be lifesaving in severe or complicated cases, such as in this patient.

In conclusion, this case report discusses the diagnostic and therapeutic approach to an unusual hemorrhagic presentation in a newborn. This paper aims to provide information on such a rare pathology to help improve diagnosis and treatment. More research is needed to develop guidelines for managing post-circumcision bleeding in newborns with coagulopathy.
